# A Coupled Double-Layer Electrical Impedance Tomography-Based Sensing Skin for Pressure and Leak Detection

**DOI:** 10.3390/s24134134

**Published:** 2024-06-26

**Authors:** Petri Kuusela, Aku Seppänen

**Affiliations:** Department of Technical Physics, University of Eastern Finland, 70210 Kuopio, Finland; aku.seppanen@uef.fi

**Keywords:** electrical impedance tomography, sensing skin, pressure sensing, leak detection

## Abstract

There is an extensive need for surface sensors for applications such as tactile sensing for robotics, damage and strain detection for structural health monitoring and leak detection for buried structures. One type of surface sensor is electrical impedance tomography (EIT)-based sensing skins, which use electrically conductive coatings applied on the object’s surface to monitor physical or chemical phenomena on the surface. In this article, we propose a sensing skin with two electrically coupled layers separated by an insulator. Based on electrical measurements, the spatial distribution of the electrical coupling between the layers is estimated. This coupling is sensitive to both the pressure distribution on the surface and water entering between the layers through a leak. We present simulations and experimental studies to evaluate the feasibility of the proposed method for pressure sensing and leak detection. The results support the feasibility of the proposed method for both of these applications.

## 1. Introduction

The need for surface sensors is prevalent in many fields. The quickly developing field of robotics requires novel smart surface sensors for pressure and tactile sensing, e.g., for human–robot interactions and safety concerns [1,2,3,4,5,6,7,8,9,10,11,12,13,14,15,16]. Similar sensing is required in some biomedical applications [2]. Sensing touch and pressure have been approached by several methods, such as grid-based capacitive sensors [14], functional materials [15], optical wave guides [16] and arrayless supercapacitive sensors [17]. In structural health monitoring (SHM), the structural integrity and lifetime expectation is analyzed based on continuous measurements. While these measurements are not necessarily performed on surface sensors, there are many promising candidates for that, such as fiber optic [18] or piezoresistive sensors [19] and strain sensors [20]. SHM is also used for leak detection in buried structures and monitoring geomembrane integrity in landfills or sewage ponds [21,22,23].

One of the latest developments in smart surface sensing is the so-called sensing skin based on electrical impedance tomography (EIT). It is an imaging method where the conductivity distribution of a target is estimated based on current and voltage measurements on the surface of the target. EIT-based sensing skins have already been studied in detecting the cracking and fatigue of concrete [24] and steel [25], composite material damage and strain [26,27,28,29,30,31,32,33,34], pH [35], surface pressure [1,2,3,4,5,6,7,8,9,10,11,12,13,14,15,16] and temperature [36].

Although sensing skins show promise in several of the fields that they are studied in, they have certain challenges. One of them is that there can be several different physical and chemical factors contributing to the conductivity of the sensing skin, and, therefore, it may be difficult to distinguish the effect of interest. Hence, there is still need for innovative smart surface sensors for, e.g., detecting surface pressure in robotics and detecting leaks in buried structures.

In publication [37], a functional double-layer sensing skin was proposed for SHM: the two layers of the sensing skin were used separately, yet simultaneously, to distinguish different physical/chemical effects on the surface—particularly, one layer was sensitive to cracking while the other one was sensitive to both cracking and strong electrolytes, such as chlorides dissolved in water in contact with the surface. Another type of multilayer sensing skin proposed for tactile sensing is based on having a second more conductive layer that is weakly separated from the original measured surface [7,38,39]. Applied pressure introduces a contact between the two surfaces, thus effectively increasing the local conductivity of the measurement layer on the region of pressure.

In this paper, we propose a novel EIT-based sensing skin technique that is based on the use of two parallel electrically conductive layers. Similarly to the sensing skins presented in [38,39], these layers are coupled electrically, causing current to flow between the layers. In addition to the pressure on the surface, the electrical coupling is also sensitive to water entering in between the two layers. Unlike in the previous studies, we use two similar conductive layers and have electrodes attached on both of the conductive surfaces. We also measure currents flowing between electrodes of different surfaces. We formulate the mathematical model for the proposed technique and introduce its computational implementation. We study the feasibility of the proposed technique for pressure sensing and leak detection using numerical simulations and experiments.

## 2. Single-Layer EIT-Based Sensing Skin

EIT-based sensing skins consist of a conductive surface with electrodes attached to it, through which electrical currents can be injected into the surface. Traditionally, using a so-called current injection EIT device, the currents of the electrodes are set to different injection patterns and the resulting potentials are measured. In contrast, so-called potential excitation EIT devices set the potentials of the electrodes to different excitation patterns and measure the resulting currents. In many cases, both the theory and applications work similarly independent of which type of device is used. In this study, we use a potential excitation device for measurements, and hence we also formulate the theory accordingly. We use the conventional, single-layer sensing skin model in the reconstruction of the background conductivities of the two layers before they are installed in the coupled double-layer sensor system.

### 2.1. Forward Problem

The EIT measurements (whether potentials or currents are measured) are usually modeled by the complete electrode model (CEM), which consists of the following equations [40,41,42]:

(1) $$\begin{array}{cc} \nabla \cdot (\sigma (x)\nabla u(x)) & =0,\;x\in \Omega \end{array}$$

(2) $$\begin{array}{cc} u\left(x\right)+z_{l}\sigma \left(x\right)\frac{\partial u(x)}{\partial \hat{n}} & =U_{l},\;x\in \partial \Omega_{e_{l}},\;l=1,\ldots ,N_{\mathrm{el}} \end{array}$$

(3) $$\begin{array}{cc} \int_{\partial \Omega_{e_{l}}}\sigma \left(x\right)\frac{\partial u(x)}{\partial \hat{n}}dS & =-I_{l},\;l=1,\ldots ,N_{\mathrm{el}} \end{array}$$

(4) $$\begin{array}{cc} \frac{\partial u(x)}{\partial \hat{n}} & =0,\;x\in \partial \Omega \setminus \overset{N_{\mathrm{el}}}{\underset{l=1}{⋃}}\partial \Omega_{e_{l}}, \end{array}$$

where σ and *u* are the conductivity and potential inside the imaging domain Ω, $z_{l}$ is the contact impedance of the *l*:th electrode, $\hat{n}$ is the outward unit normal vector on the boundary ∂Ω, $U_{l}$ and $I_{l}$ are the potential and current on the *l*:th electrode, $\partial \Omega_{e_{l}}$ is the boundary region corresponding to the *l*:th electrode, *x* is the spatial variable and $N_{\mathrm{el}}$ is the number of electrodes in the system. Either the potentials $U_{l}$ or currents $I_{l}$ can be solved from the CEM as long as the other ones are known. In this article, as we use a potential injection device, correspondingly, we solve the currents from the CEM. In general, the potential *u* inside the domain has to be solved simultaneously with the electrode potentials or currents.

Finding an analytical solution to the CEM is generally infeasible, and hence a numerical approximation is used instead. In this article, we use a finite element method (FEM) with piecewise linear basis functions to approximate the forward model (for details, see [43]). In FEM, the imaging domain is approximated by a mesh consisting of nodes and elements. Spatially distributed quantities inside the domain are then approximated as a linear combination of basis functions $\phi_{i}$. For example, the conductivity on the domain is then approximated as

(5) $$\sigma \left(x\right)=\sum_{i=1}^{N}\sigma_{i}\phi_{i}\left(x\right),$$

where $\sigma_{i}\in R$ are coefficients of the basis functions and *N* is the number of basis functions. Using this approximation, we identify the conductivity by the vector of coefficients $\sigma_{i}$ in (5):

(6) $$\tilde{\sigma }=\left[\begin{array}{c} \sigma_{1}\\ \sigma_{2}\\ \vdots \\ \sigma_{N} \end{array}\right].$$

From the FEM, we obtain the computational model ${\tilde{I}}_{\sigma }\left(\tilde{\sigma }\right)$ mapping the conductivities to a vector containing electrode currents corresponding to all the used excitations.

### 2.2. Inverse Problem

To solve the coefficients of conductivity $\tilde{\sigma }$, a variety of methods exist. In this article, we use the Bayesian framework, in which unknown or uncertain parameter values are treated as random variables. The Bayesian framework also allows the use of prior knowledge of the measured system and the use of reference measurements. Further information can be found, e.g., in [44].

In this article, we use the *maximum a posteriori* (MAP) point as the reconstruction. The MAP point can be found by solving the following optimization problem [44]:

(7) $$\arg\min_{\tilde{\sigma }}\left\{F(\tilde{\sigma })\right\},\;F\left(\tilde{\sigma }\right)=||L\left({\tilde{I}}_{\sigma }\left(\tilde{\sigma }\right)-I_{m}\right){\left|\right|}^{2}+R\left(\tilde{\sigma }\right),$$

where $I_{m}$ contains all the measured currents from different excitations stacked into a single vector, $R(\tilde{\sigma })$ is the prior potential function (more details in Section 3.2) and *L* is the Cholesky factor of the data precision matrix.

The optimization problem (7) can be solved by many optimization algorithms. In this study, we use the Gauss–Newton (GN) method [45,46] with a line search to find the step length. For each iteration ${\tilde{\sigma }}_{i}$, the next GN iteration ${\tilde{\sigma }}_{i+1}$ with step length τ is computed by

(8) $${\tilde{\sigma }}_{i+1}\left(\tau \right)={\tilde{\sigma }}_{i}-\tau {\left(J^{T}\left({\tilde{\sigma }}_{i}\right)\Gamma^{-1}J\left({\tilde{\sigma }}_{i}\right)+H_{R}\left({\tilde{\sigma }}_{i}\right)\right)}^{-1}\left(J^{T}\left({\tilde{\sigma }}_{i}\right)\Gamma^{-1}\left(\tilde{I}\left({\tilde{\sigma }}_{i}\right)-I_{m}\right)+\nabla R\left({\tilde{\sigma }}_{i}\right)\right),$$

where *J* is the Jacobian of $\tilde{I}\left(\tilde{\sigma }\right)$ with regard to $\tilde{\sigma }$ and $H_{R}$ and ∇R are the Hessian and the gradient of *R*. The step length τ is chosen optimally with respect to the objective function

(9) $$\tau =\arg\min_{\tau^{\prime }}\left\{F\left({\tilde{\sigma }}_{i+1}\left(\tau^{\prime }\right)\right)\right\}.$$

## 3. A Coupled Double-Layer Sensing Skin

**Figure 1:**
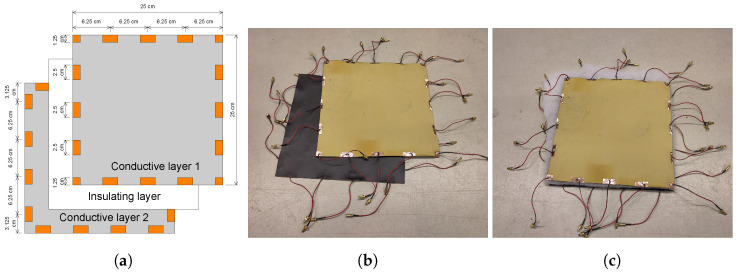
(**a**) A schematic diagram showing the layers of the sensing skin and locations of electrodes. The electrodes are drawn in copper brown. (**b**) The two conductive layers of the sensing skin without the insulating middle layer. The black side of the layer is the conductive paint and the yellow side shows the rubber base of the layer. (**c**) The assembled sensing skin with the insulating layer in the middle.

The double-layer sensing skin presented in this paper consists of two conductive surfaces separated by an insulating layer, as depicted in Figure 1. Although the middle layer is insulating, some current will pass through it as displacement current since alternating potential difference is applied to the electrodes. Alternatively, in case of leak detection, water can form a conductive pathway between the layers. We use single-electrode excitations, i.e., one electrode at a time is excited to a higher potential, while the others remain grounded. Currents passing through the grounded electrodes are then measured.

In this paper, we model the double-layer sensing skin as two two-dimensional conductive surfaces modeled by the CEM and a coupling between the surfaces. More specifically, the coupling is the conductance per area, and thus, together with the local potential difference between the layers, it determines the current density flowing between the layers. This coupling strengthens if water leaks into the space between the layers (a conductive pathway) or if applied pressure pushes the layers closer to each other (increased local capacitance). For the purely capacitive case, the coupling can be computed by assuming that the layers form a parallel plate capacitor with possibly spatially varying distance d(x) between the layers. For a small area *A* having d(x) approximately constant, the capacitance between the layers for that area is

(10) $$C=\epsilon \frac{A}{d(x)},$$

where ϵ is the electrical permittivity of the insulator between the layers. With signal angular frequency ω, this leads to conductance

(11) $$G=j\omega C=j\omega \epsilon \frac{A}{d(x)},$$

where *j* is the imaginary unit. From this, we obtain

(12) $$c\left(x\right)=\frac{G}{A}=\frac{j\omega \epsilon }{d(x)}.$$

In this article, we neglect the phase of the coupling, i.e., we use only the absolute value of the coupling computed by (12). Due to this approximation, the reconstructed couplings are to be interpreted only qualitatively.

The spatial coordinate system used for the two conductive surfaces consists of the two coordinates of the point along the surface and an additional index distinguishing the two layers. Therefore, the domain for potential *u* and conductivity σ is $\Omega \subset R^{2}\times \left\{1,2\right\}$. On the other hand, the coupling between the layers is shared by the two surfaces, and thus has the domain $\Omega_{c}\subset R^{2}$. We approximate the areas under the attached electrodes to remain in the same potential as the electrode, and therefore do not consider these areas as a part of the domain Ω, as the local potential *u* is not solved from the CEM there. However, the area under an electrode on the surface that the electrode is not attached on is part of domain Ω.

**Figure 2:**
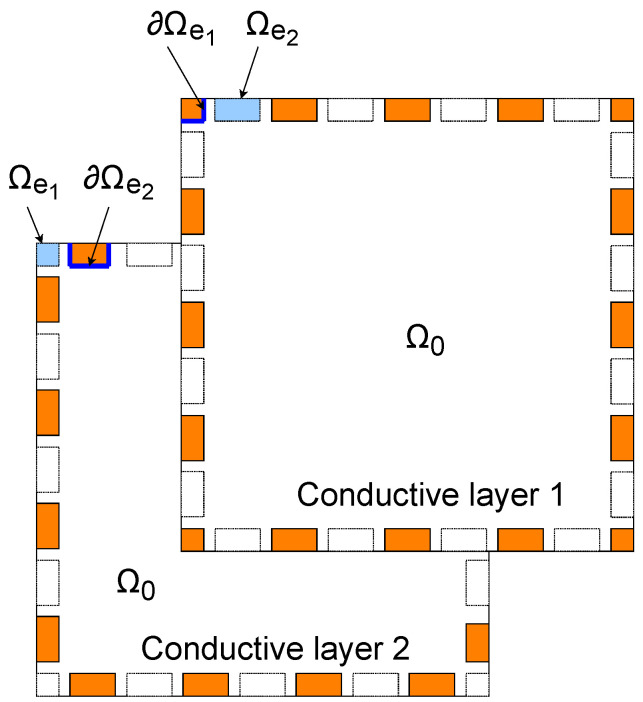
A schematic diagram of the imaging domain of the double-layer sensing skin. $\Omega_{0}$ is the area common for both layers. $\Omega_{e_{l}}$ is the area under electrode $e_{l}$ on the surface that the electrode is not attached to and $\partial \Omega_{e_{l}}$ is the domain of the boundary corresponding to electrode $e_{l}$. Electrodes are drawn in copper color on the layer that they are attached to. Labels for electrodes 1 and 2 are shown as examples, with blue highlights used to differentiate where the boundary and where the area of the electrode are meant.

We notate the area where neither of the surfaces has electrodes as $\Omega_{0}$, i.e., $\Omega_{0}=\left\{(x,i)\in \Omega \;|\;(x,j)\in \Omega \;\forall \;j=1,2\right\}$. In addition, the area under electrode *l* on the surface that the electrode is not attached to is notated as $\Omega_{e_{l}}$. Similarly as before, the boundary corresponding to electrode *l* is denoted by $\partial \Omega_{e_{l}}$. The subdomains are illustrated in Figure 2. It is worth noting that $\Omega =\Omega_{0}\cup {⋃}_{l=1}^{N_{\mathrm{el}}}\Omega_{e_{l}}$ and $\Omega_{c}=\left\{x\in R^{2}\;|\;\exists \;j\in \left\{1,2\right\}\;\mathrm{such}\mathrm{that}\;\left(x,j\right)\in \Omega \right\}$.

Adding the coupling into the CEM requires modifying Equation (1), which is the conservation of currents in the continuum. In the coupled double-layer system, the current on each layer does not have to be conserved, but instead, any current sink on one layer has to be matched by a current source on the other layer, and vice versa. Thus, the total current is conserved, although, on the 2D surfaces, it is not.

Adding these sinks and sources to Equation (1) leads to the set of equations

(13) $$\begin{array}{cc} \nabla \cdot (\sigma (x,1)\nabla u(x,1)) & =c\left(x\right)\left(u(x,2)-u(x,1)\right),\;\left(x,1\right)\in \Omega_{0}, \end{array}$$

(14) $$\begin{array}{cc} \nabla \cdot (\sigma (x,2)\nabla u(x,2)) & =c\left(x\right)\left(u(x,1)-u(x,2)\right),\;\left(x,2\right)\in \Omega_{0}, \end{array}$$

where u(x,i) and σ(x,i) are the potential and conductivity, respectively, at point (x,i) and c(x) is the coupling at point *x*. The coupling c(x), together with the potential difference in the two layers, determines the current density flowing between the surfaces. In Equations (13) and (14), we consider only the area without electrodes on either surface. Wherever one of the surfaces has an electrode, the potential function *u* is not defined, and instead we use the potential of the electrode

(15) $$\nabla \cdot \left(\sigma \left(x,i\right)\nabla u\left(x,i\right)\right)=c\left(x\right)\left(U_{l}-u\left(x,i\right)\right),\;\left(x,i\right)\in \Omega_{e_{l}},\;l=1,\ldots ,N_{\mathrm{el}},$$

where $\Omega_{e_{l}}$ is the area under electrode *l* on the surface that the electrode is not attached to.

Equations (13)–(15) can be combined into a single equation by first defining a function for the potential on the other layer

(16) $$u_{2}\left(x,i\right)=\left\{\begin{array}{cc} u(x,2),\; & \left(x,i\right)\in \Omega_{0},\;i=1\\ u(x,1),\; & \left(x,i\right)\in \Omega_{0},\;i=2\\ U_{l},\; & \left(x,i\right)\in \Omega_{e_{l}}. \end{array}\right.$$

Hence, we can write the CEM extended for the coupled double-layer sensing skin:

(17) $$\begin{array}{cc} \nabla \cdot (\sigma (x,i)\nabla u(x,i)) & =c\left(x\right)\left(u_{2}\left(x,i\right)-u\left(x,i\right)\right),\\ & \;(x,i)\in \Omega , \end{array}$$

(18) $$\begin{array}{cc} u\left(x,i\right)+z_{l}\sigma \left(x,i\right)\frac{\partial u(x,i)}{\partial \hat{n}} & =U_{l}, \end{array}$$

$$\begin{array}{c} \;\left(x,i\right)\in \partial \Omega_{e_{l}},\;l=1,\ldots ,N_{\mathrm{el}} \end{array}$$

(19) $$\begin{array}{cc} \int_{\partial \Omega_{e_{l}}}\sigma \left(x,i\right)\frac{\partial u(x,i)}{\partial \hat{n}}dS+\int_{\Omega_{e_{l}}}c\left(x\right)\left(U_{l}-u\left(x,i\right)\right)d\Omega & =-I_{l},\;l=1,\ldots ,N_{\mathrm{el}} \end{array}$$

(20) $$\begin{array}{cc} \frac{\partial u(x,i)}{\partial \hat{n}} & =0,\;\left(x,i\right)\in \partial \Omega \setminus \overset{N_{\mathrm{el}}}{\underset{l=1}{⋃}}\partial \Omega_{e_{l}}. \end{array}$$

Compared to the original CEM, Equation (19) is also modified to take into account the current sources and sinks on $\Omega_{e_{l}}$ that contribute to the measured currents as well.

### 3.1. Forward Model Solution

In this paper, we express the coupling c(x) via a separate mesh covering the domain $\Omega_{c}$. The coupling has values $c_{i}$ at nodes $x_{i}$ of this mesh. The function c(x) is then approximated in the FEM equations by

(21) $$c\left(x\right)=\sum_{i=1}^{N}c_{i}^{\prime }\phi_{i}\left(x,j\right),\;j=1,2$$

where the coefficients $c_{i}^{\prime }$ are interpolated linearly from values $c_{i}$ based on the two meshes. Further, we notate that

(22) $$\tilde{c}=\left[\begin{array}{c} c_{1}\\ c_{2}\\ \vdots \\ c_{N} \end{array}\right],$$

and

(23) $$\tilde{z}=\left[\begin{array}{c} z_{1}\\ z_{2}\\ \vdots \\ z_{N_{\mathrm{el}}} \end{array}\right].$$

The finite element formulation for the conventional CEM is well known, but in order to take into account the modifications made to model the double-layer case, we show the full derivation in Appendix A. We derive the FEM for the potential injection case, i.e., we solve the currents from the CEM, similarly as in [43]. We emphasize that the additions presented here work with few changes also using the more conventional case of solving the potentials from the CEM. We denote the computational model of the measurements obtained from FEM by $\tilde{I}\left(\tilde{\sigma },\tilde{c},\tilde{z}\right)$, which is a mapping from the parameter space to the vectors containing currents corresponding to all used potential excitations stacked into a single vector.

The basic principles of finding the coefficients of coupling $\tilde{c}$ based on the measurements are the same as in Section 2.2. However, as the conductivity distributions of the surfaces and the contact impedances of the electrodes are not known, they are also estimated. Hence, we define a generalized estimate variable Θ, i.e., any one of $\tilde{c}$, $\tilde{\sigma }$, $\tilde{z}$ or a vector obtained by stacking any combination of these. The conductivity estimate $\tilde{\sigma }$ in the equations presented in Section 2.2 is then replaced by Θ. The reconstruction process and the constituents of Θ in each step are described in more detail in Section 4.3. For details on the simultaneous estimation of contact impedances, the reader is referred to [47,48].

### 3.2. Prior Functions

To mitigate the ill-posedness of the inverse problem, prior knowledge of the estimated distributions is used. In this study, we assume the conductivity distributions to have spatially smooth variations throughout the surfaces caused by inhomogeneities of the paint layers. On the other hand, the coupling is assumed to have a sparsely steep gradient caused by either compression of the insulating region under pressure or by water applied in between the two layers. Furthermore, both conductivity and the coupling are known to always be positive.

To promote smooth variations in the conductivity distribution, a Gaussian smoothness prior is used [49]. It has the functional form

(24) $$R_{\mathrm{sm}}\left(\sigma \right)={\left(\sigma -\sigma_{e}\right)}^{T}\Gamma_{\mathrm{sm}}^{-1}\left(\sigma -\sigma_{e}\right),$$

where $\sigma_{e}$ is the expected value of σ and $\Gamma_{\mathrm{sm}}$ determines the covariance between conductivities of all pairs of nodes in the FE mesh of each layer of the sensor. Similarly to [49], we use a Gaussian distance covariance, i.e.,

(25) $$\Gamma_{\mathrm{sm}}\left(i,j\right)=a_{\mathrm{sm}}\exp\left(-\frac{\left|\right|x_{i}-x_{j}{\left|\right|}^{2}}{2b_{\mathrm{sm}}^{2}}\right)+c_{\mathrm{sm}}\delta_{ij},$$

where $x_{i},x_{j}\in R$ are the positions of nodes *i* and *j*, $a_{\mathrm{sm}}$ is the strength of the prior, $b_{\mathrm{sm}}$ is a constant related to the length scale of the spatial smoothness, $c_{\mathrm{sm}}$ is a small stabilizing constant and $\delta_{ij}$ is the Kronecker delta. Throughout this study, we use $c_{\mathrm{sm}}=10^{-4}\times a_{\mathrm{sm}}$. To facilitate choosing the constant $b_{\mathrm{sm}}$, we define correlation length $d_{\mathrm{cor}}$ as the length where the correlation of two nodes given by (25) is 0.01. Then, for the constant $b_{\mathrm{sm}}$, we have

(26) $$b_{\mathrm{sm}}=\frac{d_{\mathrm{cor}}}{\sqrt{2\ln100}}.$$

The total variation prior is used for favouring sparse spatial changes in the coupling. The total variation prior functional is [50]

(27) $$R_{\mathrm{TV}}\left(c\right)=\sum_{k=1}^{n}A_{k}\sqrt{a_{\mathrm{TV}}\left({\left(D_{x}c\right)}_{k}^{2}+{\left(D_{y}c\right)}_{k}^{2}\right)+\beta },$$

where $A_{k}$ is the area of element *k*, *n* is the number of elements in the mesh, $a_{\mathrm{TV}}$ is a strength parameter and β is a smoothing parameter used to make the functional differentiable. $D_{x}$ and $D_{y}$ are discrete differential operators corresponding to differentiation in *x*- and *y*-directions. The strength parameter $a_{\mathrm{TV}}$ is chosen based on the procedure outlined in [50]. In short,

(28) $$a_{\mathrm{TV}}=-\frac{\ln\left(1-p\right)}{g_{\max}},$$

where *p* is the probability of having the maximal gradient norm value of $g_{\max}$ on the domain.

The positivity of estimates is enforced by a parabolic barrier function

(29) $$R_{\mathrm{pos}}\left(\Theta \right)=a_{\mathrm{pos}}\sum_{\left\{k|\Theta_{k}<\Theta_{m,k}\right\}}{\left(\Theta_{k}-\Theta_{m,k}\right)}^{2},$$

where $a_{\mathrm{pos}}$ is the strength parameter for the constraint, Θ is the generalized estimate variable and $\Theta_{m}$ is a vector containing minimum estimate values that are not penalized. Here, we allow for different minimum values for different elements of Θ because the scales of $\tilde{\sigma }$, $\tilde{c}$ and $\tilde{z}$, any of which may be in Θ, may be different.

## 4. Simulations and Experimental Studies

### 4.1. Simulation Setup

We use numerical simulations to study the overall feasibility of the proposed method. The simulations were computed using the finite element (FE) approximation of the coupled CEM detailed in Section 3.1. The imaging domain was chosen to correspond to the experimental setup, i.e., two square surfaces with side lengths of 25 cm and 16 electrodes on each layer. Measurement setups were simulated corresponding to both pressure and leak detection. For all targets, three different measurements were simulated:

1. Reference measurements of the layers separately, i.e., with zero coupling between the layers.
2. Reference measurements of the assembled sensing skin, i.e., with homogeneous coupling.
3. Measurements with the effect (i.e., applied pressure or water) in place.

Gaussian noise with a standard deviation of 0.1% of each measurement value was added to all measurements.

In the simulations, the insulating layer had a thickness of 5 mm at rest and relative permittivity $\epsilon_{r}=4$. The couplings for simulations were computed using Equation (12), except for the water ingress.

For pressure sensing, two targets (targets I and II) were simulated, both modeling pressing the distance between the layers from 5 mm down to 2 mm on a circular region having a diameter of 5 cm. Target I was simulated with the sensing skin having homogeneous conductivity of 2 $\times \;10^{-3}\;\Omega^{-1}$, while target II was simulated with the sensing skin having the conductivity distribution drawn from the smoothness prior distribution Equation (24) with parameter values $d_{\mathrm{cor}}=10$ cm, $a_{\mathrm{sm}}=1.6\times 10^{-7}\;S^{2}$ and $\sigma_{e}$ a vector containing values $2\times 10^{-3}$ S in each element. For leak detection, water ingress between the layers was simulated by an area of increased coupling (target III). Equation (12) was used for the background coupling, and the peak value on the wet area was chosen to have ten-fold coupling compared to the background. The initial conductivity distribution for this simulation was drawn from the same distribution as for target II but featured an additional increase in conductivity on the region of water ingress. For target I, contact impedances were set to a negligible value of $10^{-6}\;\Omega$. For targets II and III, the contact impedances were drawn from normal distribution N(1,1)Ω, with all values less than $10^{-6}\;\Omega$ replaced by the value $10^{-6}\;\Omega$.

A mesh of 47,639 nodes and 92,490 elements was used for the FEM simulations. The element widths varied between 0.5 mm (near the electrodes) and 16 mm (on the central regions). The conductivity was represented in a mesh of 4122 nodes and 7616 elements (element widths ranging from 3 mm to 19 mm) in total for the two surfaces, and the coupling was represented in a mesh of 790 nodes and 1478 elements (element widths approximately 10 mm). The conductivity and coupling values were linearly interpolated to the nodal points of the FE mesh.

### 4.2. Experimental Setup

For measurements, a commercial potential excitation EIT device manufactured by Rocsole Ltd. (based in Kuopio, Finland) was used. The injection frequency was chosen as 320 kHz and single-electrode excitations were used.

The two conductive surfaces were made of rubber sheets painted with conductive paint. The paint was a mixture of rubber coating (Maston RUBBERcomp) and graphite powder (CRETACOLOR) added for conductivity in a mix ratio of 1:10 by weight. Electrodes were manufactured of copper tape, which was attached to the rubber sheet with the pre-applied adhesive before applying the paint. The pieces of copper tape were longer than the modeled electrode so that the rest of the piece of tape was turned on the back side of the rubber sheet. Short pieces of wire, with SMB connectors on the other end, were soldered on the copper tapes on the back side. The conductive paint was applied by brush so that the layer of paint covers the entire front surface of each rubber sheet, including the parts of the attached electrodes on the front side. Two layers of conductive paint were applied to obtain a more even coating. The first layer was left to dry overnight before applying the second layer of paint. The insulating layer between the conductive surfaces was made of polyester padding when sensing pressure, and polymer foam when sensing water. The sensing skin was assembled by placing the insulating layer between the rubber sheets, both of the rubber sheets having their front sides, i.e., the conductive surfaces, facing the insulating layer. The layers were not attached together but were only placed resting on top of each other in order to facilitate reusability with different insulating materials.

We note that the choice of the materials used in this experiment is not necessarily optimal for the final applications, but they were chosen to facilitate the laboratory experiments. For example, instead of using rubber sheets for the conductive layers for leak detection, a water-transparent material, such as conductive fabric (e.g., the one used in [9]), may be desirable. Otherwise, a rupture in one of the conductive layers is required for the water to enter the insulating layer and be detected.

A similar set of three measurements was taken as described in Section 4.1 for the simulations, except measurement set 1 (layers measured separately), which was taken only once at the beginning and used as the reference data for all the targets. The second set of measurements (assembled sensing skin without pressure or water) was taken separately for each target before applying the effect.

**Figure 3:**
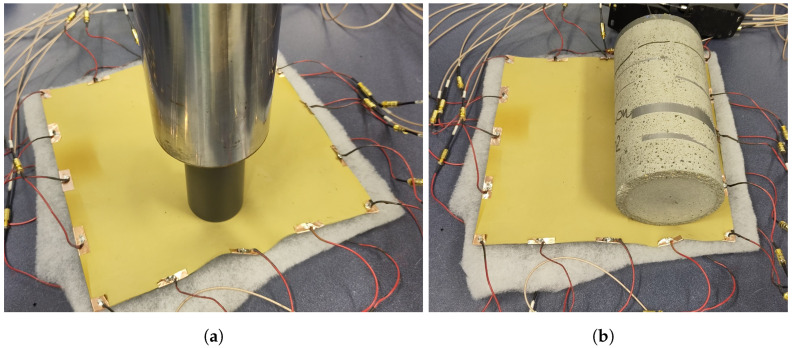
(**a**) Target IV: 11.6 kg weight and (**b**) target V: 3.4 kg weight resting on the sensing skin during measurements.

Weights of 11.6 kg and 3.4 kg were placed separately on the sensing skin for the measurements. Target IV was a 11.6 kg weight standing on the sensing skin on a circular surface having a diameter of 4.5 cm. Target V was a 20 cm tall circular cylinder (diameter 10 cm) weighing 3.4 kg resting on its side (see Figure 3). For target VI, 10 ml of water was injected in between the layers for the leak detection measurements.

### 4.3. Reconstruction

The reference measurements can be used in several ways to mitigate some of the modeling errors present in the system. The reconstruction process used in this study is detailed in this section. During the whole process, the noise of the forward model is assumed to consist of two additive Gaussian components. The first component, a baseline noise, has the same standard deviation $s_{1}$ for each measured value, with $s_{1}$ being 0.01% of the difference in maximum and minimum measured current. The second component has a standard deviation of 3% of each measured value. We note that the actual measured noise is well below this level, but these values try to approximately include the possible modeling errors as well. For further mitigating the effects of modeling errors, the Bayesian approximation error approach [51,52] could be applied, but it is left outside the scope of this paper.

To use the reference data, solving the inverse problem is divided into three steps:

1. From measurements made of the two layers separately, their initial conductivity distributions ${\tilde{\sigma }}_{0}$ and the contact impedances ${\tilde{z}}_{0}$ of electrodes are estimated as in single-layer EIT described in Section 2.
2. From measurements, where the layers were combined, but no pressure or water had yet been applied, a homogeneous estimate $c_{0}$ of the coupling is computed, using the conductivity distribution ${\tilde{\sigma }}_{0}$ and contact impedances ${\tilde{z}}_{0}$ estimated in step 1. Using the estimate $c_{0}$, a correction to the measurements is computed as specified in reference [24].
3. From measurements after applying pressure or water, the coupling distribution is estimated together with re-estimation of the conductivity distribution.

The conductivity distribution is re-estimated at the final step in addition to the coupling distribution since applying pressure or water is also presumed to change the conductivity distribution. The estimations in steps 1 and 3 are performed using the GN algorithm outlined in Section 2.2. In step 2, the estimation is simple, as the homogeneous estimate is characterized by a single parameter. The estimation of contact impedances is performed as presented in [47,48].

In step 1, the generalized estimate variable $\Theta ={\left[{\tilde{\sigma }}^{T}\;{\tilde{z}}^{T}\right]}^{T}$. The prior potential function for this step is

(30) $$R\left(\Theta \right)=R_{\mathrm{sm}}\left(\tilde{\sigma }\right)+R_{\mathrm{pos}}\left(\Theta \right),$$

with parameters $d_{\mathrm{cor}}=5$ cm, $a_{\mathrm{sm}}=4\times 10^{-10}\;S^{2}$, $\sigma_{e}$ a vector containing values $2\times 10^{-3}$ S in each element, $\Theta_{m,k}=10^{-5}$ S and $a_{\mathrm{pos}}=10^{5}\;S^{-2}$ for conductivity values and $\Theta_{m,k}=10^{-5}\;\Omega$ and $a_{\mathrm{pos}}=10^{3}\;\Omega^{-2}$ for contact impedance values.

In step 3, the generalized estimate variable $\Theta ={\left[{\tilde{\sigma }}^{T}\;{\tilde{z}}^{T}\right]}^{T}$ and the prior potential function is

(31) $$R\left(\Theta \right)=R_{\mathrm{sm}}\left(\tilde{\sigma }\right)+R_{\mathrm{TV}}\left(\tilde{c}\right)+R_{\mathrm{pos}}\left(\Theta \right).$$

Here, the parameters used are $a_{\mathrm{sm}}={\left(0.01\times 1/n\sum_{i=1}^{n}{\tilde{\sigma }}_{0,i}\right)}^{2}$, $d_{\mathrm{cor}}=5$ cm, $\sigma_{e}={\tilde{\sigma }}_{0}$, $\beta =10^{-4}$. For reconstructions corresponding to measured data, the parameter $a_{\mathrm{TV}}$ is chosen by Equation (28), with p=0.975 and $g_{\max}={\tilde{c}}_{0}/d_{\mathrm{elem}}$, where $d_{\mathrm{elem}}$ is the average edge length of mesh elements. For reconstructions corresponding to simulated data, the same procedure is used, but the parameter value is multiplied by an additional constant of $10^{-4}$ for pressure-sensing simulations and $10^{-2}$ for leak detection simulations. The positivity constraint parameters are $\Theta_{m,k}=10^{-4}$ S and $a_{\mathrm{pos}}=10^{5}\;S^{-2}$ for conductivity values, and $\Theta_{m,k}=10^{-2}\;{\mathrm{Sm}}^{-2}$ and $a_{\mathrm{pos}}=10^{4}\;S^{-2}m^{4}$ for coupling values.

The forward problem is solved by FEM as described in Section 3.1 using a mesh with 4122 nodes and 7616 elements (element widths ranging from 3 mm to 19 mm). The coupling is defined in a mesh containing 790 nodes and 1478 elements (element widths approximately 10 mm).

## 5. Results and Discussion

### 5.1. Simulations

**Figure 4:**
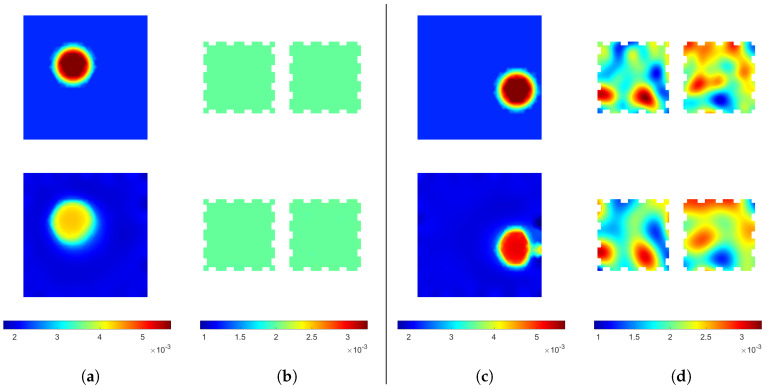
(**a**) The coupling distribution and (**b**) the conductivity distributions of target I, and (**c**) The coupling distribution and (**d**) the conductivity distributions of target II. First row: the true values. Second row: reconstructions. The conductivity distributions of the two layers are shown side by side. Couplings are in units of ${\mathrm{Sm}}^{-2}$ and conductivities in units of S.

The reconstructions computed from the pressure detection simulations (targets I and II) are shown in Figure 4. Both reconstructed locations and shapes of the inclusions are consistent with the targets within an accuracy expected from an EIT-based method. For target I, the value of coupling inside the inclusion (ca. $4.5\times 10^{-3}\;{\mathrm{Sm}}^{-2}$) does not agree with the true value (ca. $5.6\times 10^{-3}\;{\mathrm{Sm}}^{-2}$). The discrepancy of reconstructed and true values is likely caused by the total variation prior, as it penalizes stronger variations more. The homogeneous conductivity distribution of target I is reconstructed well, with the average conductivity of the reconstruction close to the true value of $2\times 10^{-3}$ S and variations in the order of 1% in the conductivity distribution of the reconstruction. For target II, the reconstructed coupling value inside the inclusion (ca. $5.2\times 10^{-3}\;{\mathrm{Sm}}^{-2}$) agrees with the true value (ca. $5.6\times 10^{-3}\;{\mathrm{Sm}}^{-2}$) better than for target I, but the shape of the inclusion is more distorted. The distortion of the shape may be caused by the proximity of the boundary and electrodes, increasing the modeling error caused by using a different FE mesh for the reconstruction than that used for the data simulation. The reconstructed conductivity distribution of target II shows all the features of the target within the expected accuracy.

These reconstructions show that, in the absence of large modeling errors, the size and location of single areas of pressure can be reconstructed fairly accurately even with inhomogeneous background conductivity of the sensing skin. The robustness of quantifying the value of coupling inside an inclusion has to be improved for applications requiring the absolute value, e.g., for quantifying the applied pressure on a surface. For many applications, however, the location of the applied pressure is enough, and, for those applications, the proposed method is viable.

**Figure 5:**
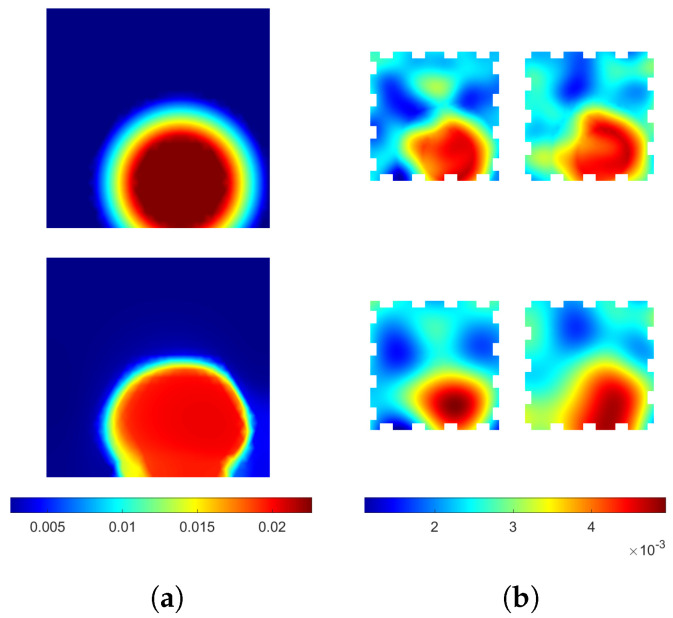
(**a**) The coupling distribution and (**b**) the conductivity distributions of target III. First row: the true values. Second row: reconstructions. The conductivity distributions of the two layers are shown side by side. Couplings are in units of ${\mathrm{Sm}}^{-2}$ and conductivities in units of S.

In Figure 5, the reconstructions corresponding to water ingress simulation (target III) are shown. The area of water ingress is depicted well in the coupling reconstruction, with the numerical value (ca. $2.1\times 10^{-2}\;{\mathrm{Sm}}^{-2}$) being quite close to correct (ca. $2.4\times 10^{-2}\;{\mathrm{Sm}}^{-2}$). In addition, the conductivities are reconstructed well within the accuracy expected, with also the increased conductivity in the area of water ingress visible.

This evidence supports the feasibility of leak detection by the proposed double-layer sensing skin. The minor discrepancies between the reconstructed coupling and the true distribution are irrelevant for the application of leak detection.

### 5.2. Experimental Results

**Figure 6:**
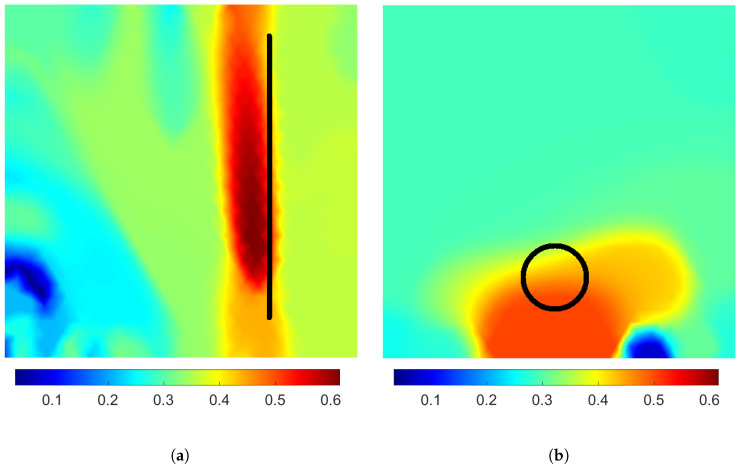
Reconstructed coupling distributions computed from measured data. (**a**) The black line corresponds to the central line of contact of the 3.4 kg cylinder resting on its side. (**b**) The black circle corresponds to the bottom surface of the 11.6 kg weight. The units are in ${\mathrm{Sm}}^{-2}$.

The experimental results of pressure detection (targets IV and V) are shown in Figure 6. In this figure, only the reconstructions of the coupling distribution are represented. Both reconstructions show an increased coupling on or close to the correct area. Both reconstructions feature some artifacts of decreased coupling as well. As the applied pressure should not cause a decrease in coupling, these artifacts are easily distinguished from the locations of actual applied pressure.

The reconstruction of target IV in Figure 6a shows the elongated shape of the pressure distribution well. However, the area of increased coupling is reconstructed as continuing through the sensing skin, from one boundary to another, whereas the edges of the object resting on the surface are 2.5 cm from both boundaries. This error is likely caused by the surface deforming further than the exact location of applied pressure.

From the reconstruction of target V in Figure 6b, the size and shape of the inclusion cannot be inferred. The spreading of the increased coupling to a larger area is likely caused by the surface deforming on a larger area than the applied pressure. This deformation may also include wrinkling of the surface, which may explain some artifacts even having decreased electrical coupling. Another possible cause of significant error is the alteration in contact impedances caused by the stretching and wrinkling of the conductive sheets under the electrodes.

Although the proposed method seems prone to having some artifacts and inaccuracies in the location and shape of the observed distribution, the results support the feasibility of detecting the location of applied pressure using the proposed method. In this study, we used weights in the order of several kilograms, but we emphasize that the weight range that the sensing skin is sensitive to can be tuned by choosing the materials of conductive and insulating layers.

**Figure 7:**
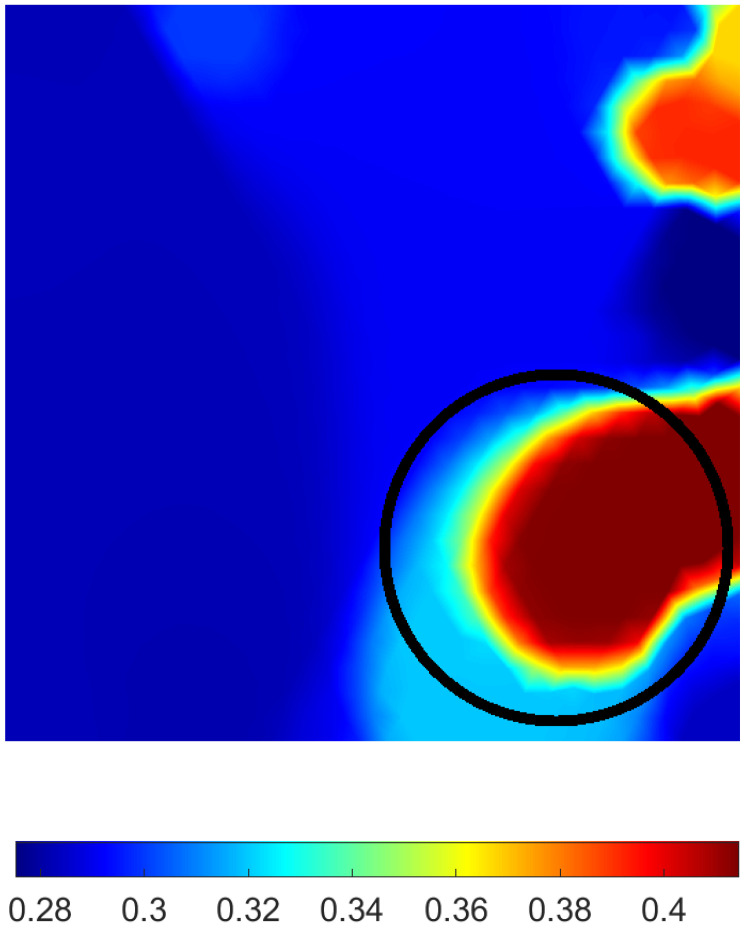
Reconstructed coupling distribution computed from measured data corresponding to water injected between the layers. The black circle marks the approximate location of water injection. The units are in ${\mathrm{Sm}}^{-2}$.

In Figure 7, the reconstructed coupling distribution corresponding to the water ingress (target VI) is shown. The reconstruction features increased coupling coinciding well with the approximate location of the water ingress. However, in addition, there is another region of increased coupling with no clear explanation. This may be caused, for example, by variations in contact impedances or by slight bending of the edges of the conductive layers. Despite the artifact, the location of water ingress is clearly visible, and thus the results support the viability of using the proposed method for leak monitoring.

## 6. Conclusions

In this article, we studied the feasibility of using EIT-based electrically coupled double-layer sensing skin for pressure sensing and leak detection. The aim of the method is to reconstruct the spatial distribution of the electrical coupling between the two layers. This distribution contains information of possible external pressure pushing the layers closer to each other, or a conductive path of water formed between the layers.

The feasibility of the proposed method for pressure sensing and leak detection was tested on both simulated and measured data. The reconstructions corresponding to simulated data localized both the applied pressure and the ingress of water, with slight distortions in the shape or the reconstructed electrical coupling value. The reconstructions from measured data had some artifacts, but the approximate locations of the applied pressure or water leak could still be inferred from the reconstructions. While the reconstruction from a circular pressure distribution failed to show the shape of the applied pressure, the reconstruction of a line-like target showed the elongated shape of the applied pressure.

The results of both the simulations and the experimental studies support the feasibility of the proposed method for pressure sensing and leak detection. Hence, the proposed method may be used in applications such as tactile sensing in robotics and leak monitoring in buried structures.

## Data Availability

The original data presented in the study are openly available in Github repository PetriKuusela/DoubleLayerSensingSkin at https://github.com/PetriKuusela/DoubleLayerSensingSkin, accessed on 13 May 2024.

## Abbreviations

The following abbreviations are used in this manuscript:

EIT	Electrical impedance tomography
SHM	Structural health monitoring
CEM	Complete electrode model
FEM	Finite element method
MAP	Maximum a posteriori
GN	Gauss–Newton
FE	Finite element

## Appendix A. FEM Formulation for Coupled Double-Layer Sensing Skin

In this appendix, we present the derivation of the finite element method formulation for the coupled double-layer sensing skin. The derivation follows along the lines of previous similar derivations, such as [41,43]. For the derivation, in addition to requiring (17)–(20), i.e.,

(A1) $$\begin{array}{cc} \nabla \cdot (\sigma (x,i)\nabla u(x,i)) & =c\left(x\right)\left(u_{2}\left(x,i\right)-u\left(x,i\right)\right),\;\left(x,i\right)\in \Omega , \end{array}$$

(A2) $$\begin{array}{cc} u\left(x,i\right)+z_{l}\sigma \left(x,i\right)\frac{\partial u(x,i)}{\partial \hat{n}} & =U_{l},\;\left(x,i\right)\in \partial \Omega_{e_{l}},\;l=1,\ldots ,N_{\mathrm{el}} \end{array}$$

(A3) $$\begin{array}{cc} \int_{\partial \Omega_{e_{l}}}\sigma \left(x,i\right)\frac{\partial u(x,i)}{\partial \hat{n}}dS+\int_{\Omega_{e_{l}}}c\left(x\right)\left(U_{l}-u\left(x,i\right)\right)d\Omega & =-I_{l},\;l=1,\ldots ,N_{\mathrm{el}} \end{array}$$

(A4) $$\begin{array}{cc} \frac{\partial u(x,i)}{\partial \hat{n}} & =0,\;\left(x,i\right)\in \partial \Omega \setminus \overset{N_{\mathrm{el}}}{\underset{l=1}{⋃}}\partial \Omega_{e_{l}}, \end{array}$$

we require conservation of currents on the electrodes

(A5) $$\sum_{l=1}^{N_{\mathrm{el}}}I_{l}=0.$$

To derive the variational form, we multiply both sides of (A1) by a test function v:Ω→R and integrate over the domain Ω to obtain

(A6) $$\int_{\Omega }v\left(x,i\right)\nabla \cdot \left(\sigma \left(x,i\right)\nabla u\left(x,i\right)\right)\;d\Omega =\int_{\Omega }v\left(x,i\right)c\left(x\right)\left(u_{2}\left(x,j\right)-u\left(x,i\right)\right)d\Omega .$$

Next, we use Green’s formula to obtain

(A7) $$\begin{array}{c} \int_{\partial \Omega }v\left(x,i\right)\sigma \left(x,i\right)\frac{\partial u(x,i)}{\partial \hat{n}}\;dS-\int_{\Omega }\sigma \left(x,i\right)\nabla u\left(x,i\right)\cdot \nabla v\left(x,i\right)\;d\Omega \;\;\;\;\\ =\int_{\Omega }v\left(x,i\right)c\left(x\right)\left(u_{2}\left(x,i\right)-u\left(x,i\right)\right)\;d\Omega . \end{array}$$

Next, the boundary integral is separated into electrode and non-electrode boundaries and boundary conditions (A2) and (A4) are used. As a result, we obtain

(A8) $$\begin{array}{c} \sum_{l=1}^{N_{\mathrm{el}}}\frac{1}{z_{l}}\int_{\partial \Omega_{e_{l}}}v\left(x,i\right)\left(u\left(x,i\right)-U_{l}\right)\;dS-\int_{\Omega }\sigma \left(x,i\right)\nabla u\left(x,i\right)\cdot \nabla v\left(x,i\right)\;d\Omega \;\;\\ =\int_{\Omega }v\left(x,i\right)c\left(x\right)\left(u_{2}\left(x,i\right)-u\left(x,i\right)\right)\;d\Omega . \end{array}$$

Now, we split the integral containing $u_{2}\left(x,i\right)$ into electrode and non-electrode parts using (16) to obtain

(A9) $$\begin{array}{c} \sum_{l=1}^{N_{\mathrm{el}}}\frac{1}{z_{l}}\int_{\partial \Omega_{e_{l}}}v\left(x,i\right)\left(u\left(x,i\right)-U_{l}\right)\;dS-\int_{\Omega }\sigma \left(x,i\right)\nabla u\left(x,i\right)\cdot \nabla v\left(x,i\right)\;d\Omega \;\;\\ =\int_{\Omega_{0}}v\left(x,i\right)c\left(x\right)\left(u_{2}\left(x,i\right)-u\left(x,i\right)\right)\;d\Omega +\sum_{l=1}^{N_{\mathrm{el}}}\int_{\Omega_{e_{l}}}v\left(x,i\right)c\left(x\right)\left(U_{l}-u\left(x,i\right)\right)\;d\Omega . \end{array}$$

Next, we introduce a test vector $\iota \in R^{N_{\mathrm{el}}}$. By multiplying (A2) by $\iota_{l}$ and integrating over electrode *l*, we obtain

(A10) $$\begin{array}{c} \int_{\partial \Omega_{e_{l}}}\left(u\left(x,i\right)+z_{l}\sigma \left(x,i\right)\frac{\partial u(x,i)}{\partial \hat{n}}\right)\iota_{l}\;dS=\int_{\partial \Omega_{e_{l}}}U_{l}\iota_{l}\;dS. \end{array}$$

To reformulate the second term on the left-hand side, we use (A3), i.e.,

(A11) $$\int_{\partial \Omega_{e_{l}}}\sigma \left(x,i\right)\frac{\partial u(x,i)}{\partial \hat{n}}\;dS=-I_{l}-\int_{\Omega_{e_{l}}}c\left(x\right)\left(U_{l}-u\left(x,i\right)\right)d\Omega$$

to obtain

(A12) $$\int_{\partial \Omega_{e_{l}}}u\left(x,i\right)\iota_{l}\;dS-\int_{\partial \Omega_{e_{l}}}U_{l}\iota_{l}\;dS-z_{l}I_{l}\iota_{l}-z_{l}\iota_{l}\int_{\Omega_{e_{l}}}c\left(x\right)\left(U_{l}-u\left(x,i\right)\right)d\Omega =0.$$

By dividing this by $z_{l}$ and then summing over all electrodes, we obtain

(A13) $$\begin{array}{c} \sum_{l=1}^{N_{\mathrm{el}}}\frac{1}{z_{l}}\int_{\partial \Omega_{e_{l}}}u\left(x,i\right)\iota_{l}\;dS-\sum_{l=1}^{N_{\mathrm{el}}}\frac{1}{z_{l}}\int_{\partial \Omega_{e_{l}}}U_{l}\iota_{l}\;dS-\sum_{l=1}^{N_{\mathrm{el}}}I_{l}\iota_{l}\\ -\sum_{l=1}^{N_{\mathrm{el}}}\iota_{l}\int_{\Omega_{e_{l}}}c\left(x\right)\left(U_{l}-u\left(x,i\right)\right)d\Omega =0. \end{array}$$

Now, by summing (A9) and (A13), we obtain the variational form

(A14) $$\begin{array}{c} \sum_{l=1}^{N_{\mathrm{el}}}\frac{1}{z_{l}}\int_{\partial \Omega_{e_{l}}}u\left(x,i\right)\left(v\left(x,i\right)-\iota_{l}\right)\;dS-\int_{\Omega }\sigma \left(x,i\right)\nabla u\left(x,i\right)\cdot \nabla v\left(x,i\right)\;d\Omega +\sum_{l=1}^{N_{\mathrm{el}}}I_{l}\iota_{l}\\ \;\;\;\;\;\;\;\;\;\;+\sum_{l=1}^{N_{\mathrm{el}}}\int_{\Omega_{e_{l}}}c\left(x\right)u\left(x,i\right)\left(v\left(x,i\right)-\iota_{l}\right)d\Omega +\int_{\Omega_{0}}c\left(x\right)\left(u\left(x,i\right)-u_{2}\left(x,i\right)\right)v\left(x,i\right)\;d\Omega \\ \;\;\;\;\;\;\;\;\;\;\;\;\;\;\;\;\;\;\;\;\;\;\;\;\;\;=\sum_{l=1}^{N_{\mathrm{el}}}\frac{1}{z_{l}}\int_{\partial \Omega_{e_{l}}}U_{l}\left(v\left(x,i\right)-\iota_{l}\right)\;dS+\sum_{l=1}^{N_{\mathrm{el}}}\int_{\Omega_{e_{l}}}c\left(x\right)U_{l}\left(v\left(x,i\right)-\iota_{l}\right)\;d\Omega \end{array}$$

Next, we use the finite element approximation

(A15) $$\tilde{u}\left(x,i\right)=\sum_{j=1}^{N}\alpha_{j}\phi_{j}\left(x,i\right),\;\left(x,i\right)\in \Omega ,$$

where $\tilde{u}$ is the approximated potential, *N* is the number of basis functions used and $\alpha_{j}$ are coefficients for basis functions $\phi_{j}$. In this article, we assume that none of the basis functions $\phi_{j}$ have support on both layers. In addition, we want to have basis functions satisfying (A5) for the electrode currents, so we define

(A16) $$\tilde{I}=\sum_{i=1}^{N_{\mathrm{el}}-1}\beta_{i}n_{i},$$

where $\tilde{I}$ is a vector containing the electrode currents and $\beta_{i}$ are coefficients for the basis functions $n_{i}\in R^{N_{\mathrm{el}}}$, which are of the form

(A17) $$\begin{array}{c} \begin{array}{cc} n_{1} & ={\left[1,-1,0,\ldots ,0\right]}^{T},\\ n_{2} & ={\left[1,0,-1,\ldots ,0\right]}^{T},\\ \; & \vdots \\ n_{N_{\mathrm{el}}-1} & ={\left[1,0,0,\ldots ,-1\right]}^{T}. \end{array} \end{array}$$

Now, by using the Galerkin scheme, i.e., using the basis functions $\phi_{i}$ and $n_{i}$ as the test functions *v* and $\iota_{l}$, we obtain the FEM system of equations in matrix form:

(A18) Aθ=b,

where

(A19) $$\theta =\left[\begin{array}{c} \alpha \\ \beta \end{array}\right],$$

where α and β are vectors containing the coefficients $\alpha_{i}$ and $\beta_{i}$. Matrix *A* is of the form

(A20) $$A=\left[\begin{array}{cc} B & 0\\ C & D \end{array}\right],$$

where

(A21) $$\begin{array}{ll} B(j,k) & =\int_{\Omega }\sigma \left(x,i\right)\nabla \phi_{j}\left(x,i\right)\cdot \nabla \phi_{k}\left(x,i\right)\\ & +c\left(x\right)\phi_{j}\left(x,i\right)\phi_{k}\left(x,i\right)\;d\Omega +\sum_{l=1}^{N_{\mathrm{el}}}\frac{1}{z_{l}}\int_{\partial \Omega_{e_{l}}}\phi_{j}\left(x,i\right)\phi_{k}\left(x,i\right)\;dS,\\ & \;j,k=1,\ldots ,N\;\mathrm{nodes}\;j\;\mathrm{and}\;k\;\mathrm{on}\mathrm{the}\mathrm{same}\mathrm{layer} \end{array}$$

(A22) $$\begin{array}{ll} B(j,k)= & \int_{\Omega }-c\left(x\right)\phi_{j}\left(x,i\right)\phi_{k}\left(x,i\right)\;d\Omega ,\\ & j,k=1,\ldots ,N\;\mathrm{nodes}\;j\;\mathrm{and}\;k\;\mathrm{on}\mathrm{different}\mathrm{layers}. \end{array}$$

(A23) $$\begin{array}{ll} C(j,k) & =-\sum_{l=1}^{N_{\mathrm{el}}}\frac{1}{z_{l}}\int_{\partial \Omega_{e_{l}}}\phi_{k}\left(x,i\right){\left(n_{j}\right)}_{l}\;dS-\sum_{l=1}^{N_{\mathrm{el}}}{\left(n_{j}\right)}_{l}\int_{\Omega_{e_{l}}}c\left(x\right)\phi_{k}\left(x,i\right)d\Omega \\ & =\frac{1}{z_{j+1}}\int_{\partial \Omega_{e_{j+1}}}\phi_{k}\left(x,i\right)\;dS-\frac{1}{z_{1}}\int_{\partial \Omega_{e_{1}}}\phi_{k}\left(x,i\right)\;dS\\ & +\int_{\Omega_{e_{j+1}}}c\left(x\right)\phi_{k}\left(x,i\right)\;d\Omega -\int_{\Omega_{e_{1}}}c\left(x\right)\phi_{k}\left(x,i\right)\;d\Omega \\ & \;k=1,\ldots ,N,\;j=1,\ldots ,N_{\mathrm{el}}-1 \end{array}$$

(A24) $$\begin{array}{cc} D(j,k) & =\sum_{l=1}^{N_{\mathrm{el}}}{\left(n_{j}\right)}_{l}{\left(n_{k}\right)}_{l}=\left\{\begin{array}{cc} 1, & j\neq k\\ 2, & j=k \end{array}\right.\;j,k=1,\ldots ,N_{\mathrm{el}}-1. \end{array}$$

Vector *b* in (A18) has the elements

$$b\left(j\right)=\left\{\begin{array}{cc} \sum_{l=1}^{N_{\mathrm{el}}}\left[\frac{U_{l}}{z_{l}}\int_{\partial \Omega_{e_{l}}}\phi_{j}\left(x,i\right)\;dS+\int_{\Omega_{e_{l}}}c\left(x\right)U_{l}\phi_{j}\left(x,i\right)\;d\Omega \right],\; & 1\leq j\leq N\\ -\sum_{l=1}^{N_{\mathrm{el}}}\left[\frac{U_{l}}{z_{l}}{\left(n_{j}\right)}_{l}\left|\partial \Omega_{e_{l}}\right|+\int_{\Omega_{e_{l}}}c\left(x\right)U_{l}{\left(n_{j}\right)}_{l}\;d\Omega \right],\; & N+1\leq j\leq N+N_{\mathrm{el}}-1 \end{array}\right.$$

(A25) $$=\left\{\begin{array}{c} \sum_{l=1}^{N_{\mathrm{el}}}\left[\frac{U_{l}}{z_{l}}\int_{\partial \Omega_{e_{l}}}\phi_{j}\left(x,i\right)\;dS+\int_{\Omega_{e_{l}}}c\left(x\right)U_{l}\phi_{j}\left(x,i\right)\;d\Omega \right],\;1\leq j\leq N\\ \begin{array}{c} \frac{U_{j+1}}{z_{j+1}}|\partial \Omega_{e_{j+1}}|-\frac{U_{1}}{z_{1}}\left|\partial \Omega_{e_{1}}\right|+\int_{\Omega_{e_{j+1}}}c\left(x\right)U_{j+1}\;d\Omega -\int_{\Omega_{e_{1}}}c\left(x\right)U_{1}\;d\Omega ,\\ N+1\leq j\leq N+N_{\mathrm{el}}-1, \end{array} \end{array}\right.$$

where $|\partial \Omega_{e_{l}}|$ denotes the area of electrode *l*.

### Computing the Jacobian

The derivatives of the currents $\tilde{I}$ with respect to the coupling coefficients $c_{j}$ (defined in (21)) are obtained similarly as the derivatives with respect to conductivity are obtained conventionally. We start by differentiating (A18) with respect to $c_{j}$ to obtain

(A26) $$\frac{\partial }{\partial c_{j}}\left(A\theta \right)=\frac{\partial b}{\partial c_{j}}.$$

Hence,

(A27) $$\frac{\partial A}{\partial c_{j}}\theta +A\frac{\partial \theta }{\partial c_{j}}=\frac{\partial b}{\partial c_{j}}$$

(A28) $$\Leftrightarrow \frac{\partial \theta }{\partial c_{j}}=A^{-1}\left(\frac{\partial b}{\partial c_{j}}-\frac{\partial A}{\partial c_{j}}\theta \right).$$

Now, we may obtain the derivatives of the currents by differentiating (A16)

(A29) $$\frac{\partial \tilde{I}}{\partial c_{j}}=\sum_{k=1}^{N_{\mathrm{el}}-1}\frac{\partial \beta_{k}}{\partial c_{j}}n_{k}.$$

Here, the derivative on the right-hand side is computed by (A28), since

(A30) $$\beta_{k}=\theta_{N+k}.$$

Next, we compute the derivative of the FEM matrix

(A31) $$\frac{\partial A}{\partial c_{j}}=\left[\begin{array}{cc} \frac{\partial B}{\partial c_{j}} & 0\\ \frac{\partial C}{\partial c_{j}} & 0 \end{array}\right],$$

where

(A32) $$\begin{array}{c} \begin{array}{cc} \frac{\partial B}{\partial c_{j}}\left(k,l\right) & =\int_{\Omega }\phi_{j}\left(x,i\right)\phi_{k}\left(x,i\right)\phi_{l}\left(x,i\right)\;d\Omega ,\;k,l=1,\ldots ,N\;k,l\;\mathrm{on}\mathrm{the}\mathrm{same}\mathrm{layer}\\ \frac{\partial B}{\partial c_{j}}\left(k,l\right) & =\int_{\Omega }-\phi_{j}\left(x,i\right)\phi_{k}\left(x,i\right)\phi_{l}\left(x,i\right)\;d\Omega ,\;k,l=1,\ldots ,N\;k,l\;\mathrm{on}\mathrm{different}\mathrm{layers}.\\ \frac{\partial C}{\partial c_{j}}\left(k,l\right) & =\int_{\Omega_{e_{k+1}}}\phi_{j}\left(x,i\right)\phi_{l}\left(x,i\right)\;d\Omega -\int_{\Omega_{e_{1}}}\phi_{j}\left(x,i\right)\phi_{l}\left(x,i\right)\;d\Omega ,\\ \; & \;k=1,\ldots ,N,\;j=1,\ldots ,N_{\mathrm{el}}-1. \end{array} \end{array}$$

Finally, we compute the elements of the derivative of the vector *b*

(A33) $$\frac{\partial b}{\partial c_{j}}\left(k\right)=\left\{\begin{array}{cc} \sum_{l=1}^{N_{\mathrm{el}}}\int_{\Omega_{e_{l}}}\phi_{j}\left(x,i\right)U_{l}\phi_{k}\left(x,i\right)\;d\Omega ,\; & 1\leq k\leq N\\ \int_{\Omega_{e_{k+1}}}\phi_{j}\left(x,i\right)U_{k+1}\;d\Omega -\int_{\Omega_{e_{1}}}\phi_{j}\left(x,i\right)U_{1}\;d\Omega ,\; & N+1\leq j\leq N+N_{\mathrm{el}}-1, \end{array}\right.$$

and thus we have all parts required for calculating the Jacobian with respect to coupling $\tilde{c}$.
